# Time-dependent inhibition of CYP3A4-mediated midazolam metabolism by macrolide antibiotics in CYP3A4 genetic variants: Comparison with testosterone metabolism 

**DOI:** 10.5414/CP203896

**Published:** 2021-09-20

**Authors:** Takeshi Akiyoshi, Rina Naitou, Ayuko Imaoka, Mitsue Miyazaki, F. Peter Guengerich, Katsunori Nakamura, Koujiro Yamamoto, Hisakazu Ohtani

**Affiliations:** 1 Division of Clinical Pharmacokinetics, Faculty of Pharmaceutical Sciences, Keio University, Tokyo,; 2 Gunma University Graduate School of Medicine, Gunma, Japan,; 3 Department of Biochemistry, Vanderbilt University School of Medicine, Nashville, TE, USA, and; 4 Department of Pharmacy, Ryukyus University Hospital, Okinawa, Japan

**Keywords:** erythromycin, clarithromycin, mechanism-based inhibition, CYP3A4 genetic variants, time-dependent inhibition

## Abstract

Objectives: The present study aimed to evaluate the effects of CYP3A4 genetic variation on the kinetics of mechanism-based inhibition (MBI) of both inhibitors using midazolam as a substrate for comparison with our previous study, as midazolam and testosterone have different binding sites. Background: The genetic variation of cytochrome P450 (CYP) 3A4 affects MBI, expressed as the maximum inactivation rate constant (*k*_inact,max_) and the inhibitor concentration required to achieve half-maximal inactivation (*K*_I_). We previously showed, using testosterone as a substrate, that the MBI kinetics of erythromycin and clarithromycin differ among CYP3A4 variants. Materials and methods: Midazolam 1’-hydroxylation inactivation profiles of erythromycin and clarithromycin were assessed using recombinant CYP3A4.1, .2, .7, .16, and .18 expressed in *Escherichia coli*. MBI parameters were calculated from changes in the inactivation rate constant (*Δk*_obs_) by the inhibitors. Results: Both inhibitors increased *Δk*_obs_ value in a concentration- and preincubation time-dependent manner, and MBI kinetics differed among variants. Trends of differences in MBI parameters among variants were similar to those assessed using testosterone as a substrate; *K*_I_ decreased for CYP3A4.7, and *k*_inact,max_ decreased for CYP3A4.2, .7, and .16. Conclusion: The genetic variation of recombinant CYP3A4 affects the MBI profile of CYP3A4 by erythromycin and clarithromycin, while the influence of genetic variation was similarly observed regardless of substrates. Our findings are of clinical relevance because the residual enzyme activity of CYP3A4 in the presence of inhibitor was estimated to vary among genetic variants.

**What is known about this subject**

- The inhibition kinetics of mechanism-based inhibitors on cytochrome P450 (CYP) 3A4 differs among CYP3A4 genetic variants so far as assessed using testosterone as a substrate.
- Midazolam and testosterone have different binding sites.

**What this study adds**

- Erythromycin and clarithromycin inactivated midazolam 1’-hydroxylation mediated by CYP3A4 genetic variants in a time-dependent manner.
- The genetic variation of metabolic enzyme affects the inhibition profile of mechanism-based inhibitor, while the influence of genetic variation was similarly observed irrespective of which substrate was used to assess enzymatic activity.

## Introduction 

Various nonsynonymous genetic variants have been identified for cytochrome P450 (CYP) 3A4, one of the major drug-metabolizing enzymes [1]. The effects of these genetic variations on the metabolic activity of these enzymes differ among substrates. For example, the amino acid substitution Leu293Phe (CYP3A4.18) decreases midazolam metabolism [2] but increases testosterone metabolism [3]. This phenomenon can be explained by the difference in the manner of interaction between the substrates and the CYP3A4 catalytic site. Indeed, three distinct binding domains around the CYP3A4 catalytic site [4, 5, 6, 7, 8] and the distinct or preferential binding domain for each substrate [8, 9] have been proposed. Moreover, the substrate binding site is considered to also determine the metabolic pathways of certain substrates; for example, the 1′- and 4-hydroxylation pathways of midazolam are attributable to its independent binding sites in CYP3A4 catalytic sites [8, 10, 11, 12]. Hackett [13] showed in a simulation study that Ser119 forms a hydrogen bond with midazolam but not with testosterone, suggesting that the binding mode of substrates to the heme at the catalytic site differs among substrates. This may be the case for Leu293Phe (CYP3A4.18), which showed the opposite metabolic activities on midazolam and testosterone [2, 3]. 

The inhibition mode of macrolide antibiotics, such as erythromycin and clarithromycin, to CYP3A4 is neither competitive nor noncompetitive but time-dependent inhibition [14]. Time-dependent inhibition by erythromycin and clarithromycin is caused by the covalent binding of their reactive metabolite to the catalytic site of CYP enzyme, namely mechanism-based inhibition (MBI) [15]. MBI is of clinical importance because impaired metabolism lasts even after the MBI inhibitor is eliminated from the systemic circulation. Previous structure-based analysis showed that erythromycin can interact with multiple binding domains of CYP3A4 [16]. However, the binding domain of its intermediate metabolite on CYP3A4 remains to be investigated. We have also reported that MBI by erythromycin occurs in the metabolic reaction of wild-type (WT) CYP3A4 with midazolam, testosterone, and nifedipine in a similar kinetics [17]. However, whether the MBI kinetics of CYP3A4 genetic variants differ among substrates remains to be clarified. 

Nonsynonymous mutations of CYPs are known to affect the inhibition kinetics of both competitive and mechanism-based inhibitors [18, 19, 20]. We have assessed the MBI kinetics of CYP3A4-mediated testosterone metabolism by erythromycin and compared the kinetics among five CYP3A4 genetic variants. Our result showed that the maximum differences in the *k*_inact,max_ and *K*_I_ values were 1.8- and 5.4-fold among variants, respectively [19]. However, for other substrates that bind to different domains (e.g., midazolam), the effect of genetic variations on MBI kinetics remains to be investigated. 

The inhibition kinetic parameters obtained from the in vitro inhibition study using recombinant enzymes, which are often expressed by bacteria, are reliable and useful data for the quantitative prediction of clinical drug-drug interaction (DDI) [21, 22, 23]. In order to evaluate the effects of uncommon genetic variations, the analyses using recombinant enzymes are indispensable because human-derived samples are virtually not available for such variants. 

The aim of this study was to investigate whether the effect of genetic variations on the MBI profiles of CYP3A4 is consistent regardless of the substrate. For this purpose, we evaluated the MBI kinetics of CYP3A4 genetic variants by erythromycin and clarithromycin using midazolam as a substrate and compared the results with those of our previous study [19], in which testosterone was used as a substrate. 

## Materials and methods 

### Materials and preparation of CYP3A4 membrane fractions 

Midazolam, nitrazepam, and clarithromycin were purchased from Wako Pure Chemical Industries, Ltd. (Osaka, Japan). Erythromycin was purchased from Sigma-Aldrich Japan (Tokyo, Japan). 1′-Hydroxymidazolam was purchased from Biosciences-Discovery Labware (Tokyo, Japan). The membrane fraction of CYP3A4 (WT, .2, .7, .16, .18) expressed by *Escherichia coli* were prepared according to our previously reported method [18, 19]. All other chemicals and reagents of analytical and high-performance liquid chromatography (HPLC) grade were obtained from commercial sources. 

### Mechanism-based inhibition study 

A reaction mixture containing 300 mM K^+^ phosphate buffer (pH 7.4), 0.3 mM EDTA, CYP3A4 membrane fraction (0.0125 nmol P450/mL in incubation), and erythromycin or clarithromycin (final concentration of erythromycin: 0, 0.3, 1, 3, 10, and 30 µM for all variants; clarithromycin: 0, 0.3, 1, 3, 10, and 30 µM for rCYP3A4WT, .2, .16, and .18. and 0, 0.1, 0.3, 1, 3, and 10 µM for rCYP3A4.7) were co-incubated at 37 °C for 10 minutes (stabilization). Pre-incubation for the designated time (erythromycin: 0, 5, 15, and 30 minutes; clarithromycin: 0, 5, 15, and 30 minutes for CYP3A4 WT, .2, .7, and .18. and 0, 15, 30, and 45 minutes for CYP3A4.16) was initiated by adding the NADPH-generating system solution (final concentrations: 0.2 mM NADPH, 5 mM glucose-6-phosphate, 0.5 mM NADP, 1 U/mL glucose-6-phosphate dehydrogenase, 3 mM MgCl_2_). Next, 4 µM midazolam solution (final concentration at incubation: 2 µM) together with NADPH-generating system solution and 300 mM K^+^ phosphate buffer containing 0.3 mM EDTA were added to start the metabolic reaction for 20 minutes at 37 °C. The reaction was terminated by the addition of 450 µL of ice-cold methanol, followed by 50 µL of 0.1 µM nitrazepam as an internal standard. The mixture was then centrifuged at 3,500 ×g for 20 minutes at 4 °C, and the concentration of 1′-hydroxymidazolam in the supernatant was determined using HPLC-ultraviolet (UV) method described below. 

### Determination of 1′- hydroxymidazolam using HPLC-UV 

The HPLC system consisted of a pump (LCthe -10AD; Shimadzu, Kyoto, Japan), an UV-detector SPD-10AD (Shimadzu), and an octadecylsilyl column (Cosmosil, 5C_18_-MS-II, 4.6×250 mm, ODS; Nacalai Tesque, Kyoto, Japan). The mobile phase consisted of 50% methanol (v/v) and was pumped at a rate of 1.5 mL/min. The UV wavelength was set at 245 nm. The temperature of column oven was set at 42 °C. The concentration of 1′-hydroxymidazolam was calculated by the internal standard method using nitrazepam as an internal standard. 

### Calculation of mechanism-based inhibition parameters 

The activation rate constant (*k*_obs_) at each inhibitor concentration was calculated from the slope of the logarithm of remaining activity plotted against pre-incubation time. Next, the respective *k*_inact(0)_ value (the apparent inactivation rate constant without inhibitor) was subtracted from *k*_obs_ to obtain *Δk*_obs_. Then, Equation 1 was simultaneously fit to the *Δk*_obs_ at various concentration of inhibitor [I] using the nonlinear least-squares method to obtain the kinetic parameters, i.e., the maximal inactivation rate constant *k*_inact,max_ and the concentration of inhibitor that gives the half-maximal inactivation rate constant (*K*_I_) (Equation 1). 

### Statistical analysis 

The correlation of MBI parameters, i.e., *k*_inact,max_ and *K*_I_ values, between the substrates used for assay (i.e., midazolam and testosterone) was evaluated using Spearman’s rank correlation coefficient. The significance of the differences in *k*_inact,max_ or *K*_I_ value between the substrates used for each CYP3A4 variant was determined using Student’s t-test. A p-value of < 0.05 was considered statistically significant (IBM SPSS 25, IBM Corp., Armonk, NY, USA). 

## Results 

**Figure 1.**
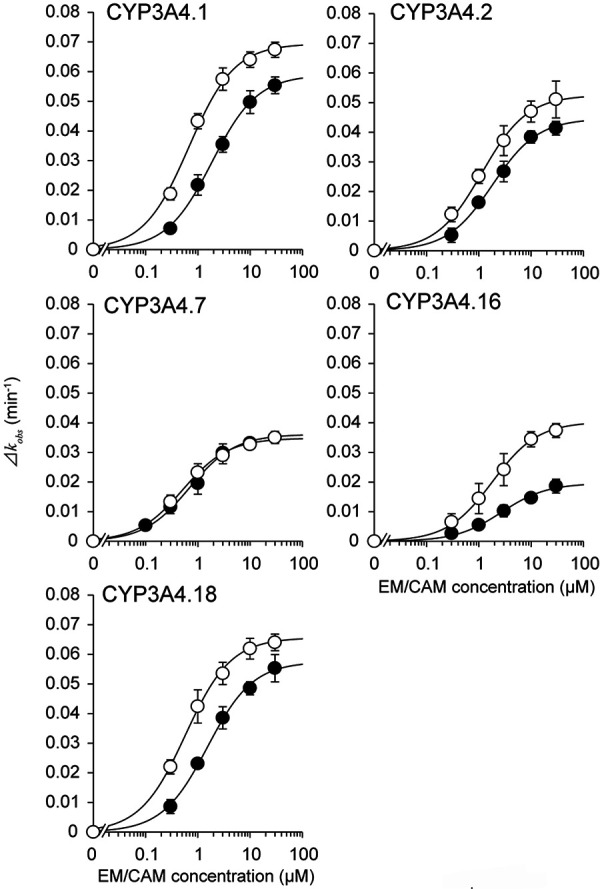
Concentration-dependent increase in the inactivation rate constant (*Δk*_obs_) of the activity of midazolam 1′-hydroxylation in five CYP3A4 genetic variants (Wt, .2, .7, .16, .18). The inactivation rate (*Δk*_obs_) was calculated as the slope of the plot of the natural logarithm of reaction velocity versus preincubation time (Supplemental Figure). Open and closed circles represent the values for erythromycin and clarithromycin, respectively. Data are presented as mean ± SD, n = 5.

Erythromycin and clarithromycin inhibited 1′-hydroxylation of midazolam by all CYP3A4 variants in a time-dependent manner ([Sec s9]). The *Δk*_obs_ value increased in an inhibitor concentration-dependent manner (Figure 1). The *k*_inact,max_ values of erythromycin for CYP3A4.2, .7, and .16 were 0.76-, 0.5-, and 0.58-fold of that for CYP3A4 WT, respectively (Table 1). The *K*_I_ values of erythromycin for CYP3A4.2 and .16 were 1.68- and 2.69-fold of that for the WT (i.e., decreased inhibitory potency), and that for CYP3A4.7 was 0.76-fold of that for the WT. Similarly, the *k*_inact,max_ values of clarithromycin for CYP3A4.2, .7, and .16 decreased (0.76-, 0.61-, and 0.33-fold vs. WT). The *K*_I_ value of clarithromycin for CYP3A4.16 was 1.45-fold of that for the WT, whereas that for CYP3A4.7 was 0.37-fold of that for the WT. 

**Figure 2.**
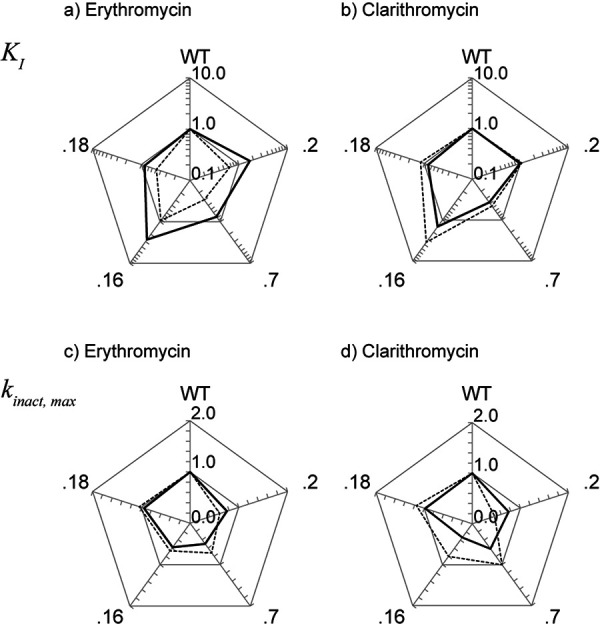
Comparison of MBI parameters between erythromycin and clarithromycin. MBI parameters, *K*_I_ (a, b) and *k*_inact,max_ (c, d), for five CYP3A4 genetic variants (Wt, .2, .7, .16, .18) are shown as the ratio to those for WT. The solid and dashed line represent the values assessed using midazolam and testosterone [19], respectively.

Figure 2 summarizes the effect of genetic variation on the MBI parameters of erythromycin and clarithromycin (shown as the ratio to those for the WT) with midazolam as a substrate, along with the results of our previous study using testosterone as a substrate [19]. The trend of the difference in inhibitory potency among CYP3A4 variants was almost consistent, regardless of the substrate used. In particular, the *K*_I_ values for CYP3A4.7 were relatively lower, whereas those for CYP3A4.16 were higher (Figure 2a, b). The *k*_inact,max_ values of both inhibitors for CYP3A4.2, .7, and .16 tended to be lower than that for the WT (Figure 2c, d). We compared the order of the parameters among variants between two studies (using midazolam vs. testosterone as substrates), and the Spearman’s rank correlation coefficient (*ρ*) for the *k*_inact,max_ and *K*_I_ values of erythromycin were 0.50 and 0.70, and those of clarithromycin were 0.30 and 0.60. 

## Discussion 

When we compare the results of the present study with those of a previous study using testosterone as a substrate (Figure 2), the trends were quite similar between the two studies; thus, we concluded that the choice of substrate may not be crucial to determine MBI kinetics, even when each substrate is known to have distinct binding sites, at least in the case of testosterone and midazolam. This phenomenon should be discussed from the viewpoint of substrate-binding. It is well known that the kinetics of midazolam 1’-hydroxylation by CYP3A4 is quite different from that of testosterone 6β-hydroxylation [8, 10, 11, 12]. Moreover, the binding mode to the catalytic site is also different between the two substrates [2, 3, 8, 10, 11, 12, 13]. On the other hand, the influence of amino acid substitutions of CYP3A4 was similar between the two substrates [24], suggesting that their metabolic processes are not fully distinct. Needless to say, the interaction of substrate with heme moiety is the common essential process of CYP-mediated metabolism, and both macrolides are metabolized by CYP3A4 to the reactive nitroso intermediate that is considered to covalently bind to heme moiety [25]. Therefore, it is conceivable that the influence of genetic variation on the MBI kinetics of macrolides, which impair heme moiety, was similarly observed irrespective of the substrate used. 

Regarding CYP3A4.7 (Gly56Asp), the *K*_I_ value was consistently lower than that for WT (i.e., increased inhibition potency), regardless of the inhibitor and substrate used. Although Gly56Asp is considered not close to heme, substitution with hydrophilic aspartic acid may have enhanced the hydrogen bond to increase the affinity of the inhibitor. Moreover, the substitution Gly56Asp has been reported to decrease the structural stability of CYP3A4 [26]; thus, this may also enhance the inactivation. 

CYP3A4.2 (Ser222Pro) was the only variant whose effect on the *K*_I_ value was different between midazolam and testosterone. This finding cannot be explained by the conventional MBI model, which assumes that the *K*_I_ value is independent of substrates. A possible reason is that the enzyme-inhibitor intermediate complex and/or “inactivated” enzyme molecule still has partial, substrate-dependent metabolic activity in some genetic variants, such as CYP3A4.2. We have previously reported that erythromycin is unable to fully inactivate the metabolic activity of CYP3A4, showing “residual” metabolic activity [17]. Therefore, certain substrates may still have access to heme, even when the intermediate metabolite has irreversibly bound to a certain domain of the enzyme. To further clarify whether the residual activity can explain the difference in the effect of Ser222Pro between midazolam and testosterone, detailed investigation of the inactivation kinetics with longer preincubation time is necessary. 

Consistent with our present result, many researchers have reported that the affinity of substrate binding for CYP3A4.16 (Thr185Ser) is lower than that for CYP3A4 WT [20, 27]. This decreased affinity can be explained by the finding of Sevrioukova and Poulos [28], in which the interaction between Thr185 in the E helix and Phe203 in the F helix plays an important role in substrate binding. Taken together, the increase in the *K*_I_ value observed in CYP3A4.16 is considered to be attributable to the decreased affinity of erythromycin and clarithromycin to heme. 

To assess the clinical relevance of the difference in the *K*_I_ values among genetic variants, we estimated the unbound concentrations of inhibitors that confer half-maximal rate of inactivation (*K*_I,u_), which is the product of *K*_I_ value and unbound fraction in microsomal preparation (f_u,mic_), and compared them with the respective maximum plasma unbound concentration in the clinical settings (C_max,u_) (Supplemental Table) [17, 29, 30]. As a result, the estimated *K*_I,u_ values, which ranged from 0.439 to 1.56 µM for erythromycin and 0.59 to 2.31 µM for clarithromycin respectively, were comparable to the respective C_max,u_ values (0.721 and 0.775 µM, respectively). Based on the static model, residual enzyme activity (*ε*) under the steady state is determined by the inactivation rate by the MBI inhibitor (*k*_inact_ ([I])) and the turnover rate in the absence of inhibitor (*k*_deg_), and calculated by using the following Equation 2. 

The *k*_inact_ ([I]) value can be calculated based on the parameters obtained in the present study and the C_max,u_ value (as the inhibitor concentration; [I]). The *k*_deg_ value of CYP3A4 is reported to be from 8.5 × 10^–5^ to 1.16 × 10^–3^ [min^–1^] [31]. Based on the above estimation, the *ε* values in CYP3A4 genetic variants were estimated to range from 3.1 to 8.2 or 0.23 to 0.63% for erythromycin and 5.6 to 21 or 0.42 to 1.9% for clarithromycin. The contribution of 1-hydroxylation by CYP3A to the overall systemic elimination of midazolam (f_m,CYP3A_) is reported to be 66.1 – 87.8% [32]) or 98% [33]). The ratio of AUC in the presence of inhibitor to that in the absence of inhibitor (AUC ratio) under the steady state can be estimated by the following equation using f_m,CYP3A_ and *ε* [34] (Equation 3), 

Assuming the f_m,CYP3A_ value to be 0.8, the AUC ratio for erythromycin was estimated to be 4.44 (*k*_deg_ assumed to be 8.5 × 0^–5^ [min^–1^]) or 4.95 (*k*_deg_ assumed to be 1.16 × 10^–3^ [min^–1^]) in subjects bearing CYP3A4 WT, which is consistent with the AUC ratio of 4.4 observed in the previous clinical study [35]. On the other hand, the AUC ratios in subjects with CYP3A4 variants were estimated to range from 3.77 to 4.45 or from 4.88 to 4.96 among four variants. The AUC ratio for clarithromycin was estimated to be 4.00 or 4.91 for subjects bearing CYP3A4 WT and rage from 2.73 to 4.08 or from 4.66 to 4.91 among subjects with CYP3A4 variants. Therefore, the differences of *K*_I_ values among variants observed in the present study can affect the extent of DDI and may be responsible for the interindividual difference in the extent of DDI, although the extent of DDI was estimated to be classified into the same category (moderate, increase in AUC of 2- to < 5-fold) based on the FDA guideline [36] in all genetic variants. 

A limitation of this study is that in addition to 1′-hydroxylation, CYP3A4 mediates the secondary metabolic pathway, i.e., 4-hydroxylation, of midazolam, for which the binding site of midazolam is considered distinct from that for the 1′-hydroxylation pathway [8, 10, 11, 12]. Lastly, although 4-hydroxylation of midazolam was not assessed because of the detection limit of 4-hydroxymidazolam, the possibility remains that the effect of genetic variations differs between metabolic pathways (and therefore between the substrates used). 

## Conclusion 

In conclusion, the MBI kinetics of erythromycin and clarithromycin on the 1′-hydroxylation of midazolam differed among five CYP3A4 genetic variants. Consistent with the results obtained using testosterone as a substrate, the *K*_I_ value of CYP3A4.7 and the *k*_inact,max_ value of CYP3A4.2, .7 and .16 were lower than the respective values of CYP3A4 WT. Our findings suggested that MBI kinetics can be affected by genetic variations of the enzyme, and the trend of this effect is considered almost consistent, regardless of the substrate. Our findings are of clinical relevance because, the *K*_I_ values of both inhibitors in genetic variants were comparable to the respective plasma concentration of both inhibitors, and the residual enzyme activity of CYP3A4 in the presence of inhibitor was estimated to vary among genetic variants. Therefore, the difference in the MBI kinetics among variants might be considered in the in vivo settings. 

## Authors’ contributions 

Participated in the research design: Akiyoshi and Ohtani. 

Preparation of CYP enzyme: Naitou, Miyazaki, Guengerich, Nakamura, and Yamamoto 

Conducted the experiments: Akiyoshi, Naitou, Imaoka, and Ohtani. 

Performed the data analysis: Akiyoshi and Naitou 

Wrote or contributed to the writing of the manuscript: Akiyoshi, Imaoka, and Ohtani. 

## Funding 

This study was supported in part by JSPS Kakenhi Grant Numbers 18K06758 (to H.O.) and US NIH grant R01 GM118122 (F.P.G.). The content is solely the responsibility of the authors and does not necessarily represent the official view of the National Institutes of Health. 

## Conflict of interest 

All authors declare that they have not received any support from any organization for the submitted work, do not have any financial relationships with any organizations that might have an interest in the submitted work, and that there are no other relationships or activities that could appear to have influenced the submitted work. 

**Table 1.** MBI kinetics parameters of erythromycin and clarithromycin on 1′-hydroxylation of midazolam by five CYP3A4 variants

	*k*_inact,max_ (min^–1^)		*K*_I_ (µM)		*k*_inact,max_/*K*_I_ (min^−1^×µM^−1^)	
	Erythromycin	Clarithromycin	Erythromycin	Clarithromycin	Erythromycin	Clarithromycin
CYP3A4 WT	0.0694 ± 0.00254*	0.0589 ± 0.00278	0.677 (0.581 ~ 0.789)*	1.85 (1.57 ~ 2.17)	0.103 ± 0.0135	0.0323 ± 0.00566
CYP3A4.2	0.0525 ± 0.00444*	0.0446 ± 0.00238*	1.14 (1.00 ~ 1.29)	1.83 (1.51 ~ 2.21)	0.0463 ± 0.00709	0.0247 ± 0.00465
CYP3A4.7	0.0348 ± 0.000808	0.0361 ± 0.00314	0.512 (0.376 ~ 0.698)	0.689 (0.464 ~ 1.02)	0.0705 ± 0.0214	0.0549 ± 0.0203
CYP3A4.16	0.0403 ± 0.000802	0.0196 ± 0.00201	1.82 (1.13 ~ 2.96)	2.69 (2.35 ~ 3.09)	0.0239 ± 0.00983	0.00734 ± 0.00135
CYP3A4.18	0.0656 ± 0.00310	0.0575 ± 0.00399*	0.580 (0.438 ~ 0.769)*	1.53 (1.27 ~ 1.86)*	0.116 ± 0.0249	0.0379 ± 0.00713

MBI = mechanism-based inhibition. Each *k*_inact,max_ value is presented as arithmetic mean ± S.D. Each *K*_I_ value is presented as geometric mean (–1S.D. ~ +1S.D.) n = 5, *p < 0.05 vs data obtained using testosterone as a substrate [19].

**Equation 1:**
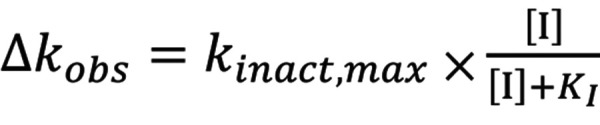
Equation 1

**Equation 2:**
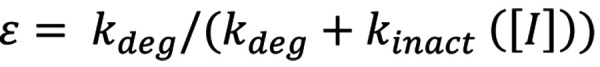
Equation 2

**Equation 3:**
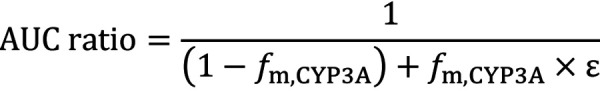
Equation 3

## Supplemental material

Supplemental material
