# Supplementary material for: Time-dependent inhibition of CYP3A4-mediated midazolam metabolism by macrolide antibiotics in CYP3A4 genetic variants: Comparison with testosterone metabolism
Source: Int J Clin Pharmacol Ther. 2021 Sep 20;59(12):745–52. doi: 10.5414/CP203896 (PMC8795987; doi:10.5414/CP203896)
Supplement: Supplemental material [file intjclinpharmacol-59-12-745-S01.pdf]

Supplemental Table. Comparison between the K_I values and clinical plasma concentrations of erythromycin and clarithromycin

	Clinical plasma concentration				*In vitro* data in the present study		
	C_{max}		$C_{max,u}$		K_I		$K_{I,u}$
	μg/mL	μM	f_u	μM	μM	$f_{u,mic}$	μM
Erythromycin	0.82[29]	1.12	0.645[29]	0.721	0.512~1.82	0.857 [17]	0.439~1.56
Clarithromycin	1.16[30]	1.55	0.5[30]	0.775	0.689~2.69	0.857	0.59~2.31

f_u; unbound fraction in human plasma, $f_{u,mic}$; estimated unbound fraction in microsomal preparation

[29] Mylan Japan Inc. "Interview Form" (a drug information booklet) of Erythromycin Tablets 100, 200 mg, 7th edition (in Japanese); 2018.

[30] Taisho Medical Inc. "Interview Form" (a drug information booklet) of Clarithromycin Tablets 100, 200 mg, 25th edition (in Japanese); 2020.

[17] Ishikawa *et al.*, Inactivation kinetics and residual activity of CYP3A4 after treatment with erythromycin. *Biopharm Drug Dispos*. 2017; 38: 420-425.

Supplemental Figure

Time-dependent inactivation of five CYP3A4 genetic variants by erythromycin or clarithromycin, as assessed by midazolam 1’-hydroxylation.

(a, f); CYP3A4.1, (b, g); CYP3A4.2, (c, h); CYP3A4.7, (d, i); CYP3A4.16, (e, j); CYP3A4.18.

The metabolic activity of CYP3A4 genetic variants, as assessed by midazolam 1′ -hydroxylation, were reduced by erythromycin and clarithromycin in a preincubation time- and concentration-dependent manner. Preincubation period was designated for 0~30 min and 0~45 min for erythromycin and clarithromycin, respectively. Data are presented as mean±SD, n=5.

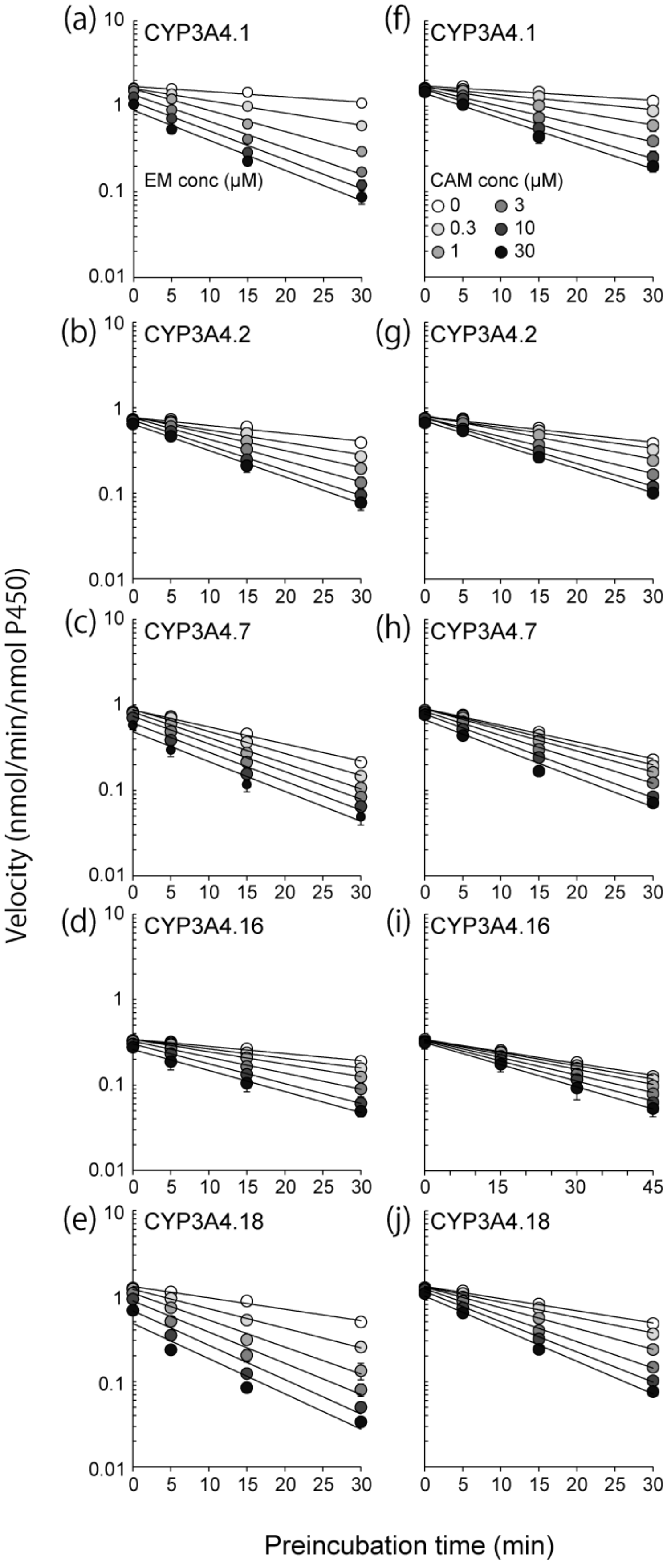

Supplemental Figure

Akiyoshi *et al.*,
